# Song Transmission as a Formal Cultural Practice

**DOI:** 10.3389/fpsyg.2021.654282

**Published:** 2021-12-08

**Authors:** Stefanie Stadler Elmer

**Affiliations:** The Schwyz University of Teacher Education and University of Zurich, Zurich, Switzerland

**Keywords:** song, cultural transmission, normativity, song grammar, cultural practice, didactics, micro-genetic analysis, aesthetic

## Abstract

From a biological point of view, the singing of songs is based on the human vocal learning capacity. It is universally widespread in all cultures. The transmission of songs is an elementary cultural practice, by which members of the older generations introduce both musico-linguistic rules and affect-regulative means to the younger ones. Traditionally, informal singing in familiar settings primarily subserves affect-regulation goals, whereas formal song transmission is embedded in various normative claims and interests, such as preserving cultural heritage and representing collective and national identity. Songs are vocal acts and abstract models that are densely structured and conform to cultural rules. Songs mirror each generations’ wishes, desires, values, hopes, humor, and stories and rest on unfathomable traditions of our cultural and human history. Framed in the emerging scientific field of didactics, I argue that research on formal song transmission needs to make explicit the norms and rules that govern the relationships between song, teacher, and pupils. I investigate these three didactic components, first, by conceptualizing song as rule-governed in terms of a grammar, with songs for children representing the most elementary musico-linguistic genre. The Children’s Song Grammar presented here is based on syllables as elements and on syntactic rules concerning timing, tonality, and poetic language. It makes it possible to examine and evaluate songs in terms of correctness and well-formedness. Second, the pupils’ learning of a target song is exemplified by an acoustical micro-genetic study that shows how vocalization is gradually adapted to the song model. Third, I address the teachers’ role in song transmission with normative accounts and provide exemplary insights into how we study song teaching empirically. With each new song, a teacher teaches the musico-linguistic rules that constitute the respective genre and conveys related cultural feelings. Formal teaching includes self-evaluation and judgments with respect to educational duties and aesthetic norms. This study of the three-fold didactic process shows song transmission as experiencing shared rule-following that induces feelings of well-formedness. I argue that making the inherent normativity of this process more explicit – here systematically at a descriptive and conceptual level – enhances the scientificity of this research domain.

## Introduction

The singing of songs builds on the human capacity for vocal production learning and our propensity to entrain to repetitive, evenly paced signals (Merker, 2006, 2012; Merker et al., 2018). Social gatherings for joint singing are a cultural practice through which the musico-linguistic lore is transmitted between generations. Joint singing is universal in all human cultures (Jordania, 2011), unless suppressed by authorities for political reasons. Producing and sharing vocal sounds that are jointly organized according to certain rules is regarded as a key issue in theorizing on the origins of music and language in anthropogony (Merker, 2005; Merker et al., 2018) and ontogeny (Stadler Elmer, 2020). Song is an elementary cultural practice accessible from infancy to old age. It may take at least three forms: a memorized vocal pattern performed by a singer who may vary and alter its form, a recording with the possibility of identical repetition, and, finally, in the form of musical notation by which the song escapes the ephemeral. As I will show, the distinctions between these three forms of song are important in the study of song transmission.

In this paper, my focus is not the evolution of song transmission but the cultural practice of intergenerational song transmission as organized in many nations’ educational systems nowadays. I examine some of the constitutive norms and rules in an attempt to render them more explicit. For this purpose, I distinguish between informal and formal transmission, and I focus on the latter in greater detail and by adopting a didactic perspective. The concept of “didactic” denotes an emerging scientific field in Europe that systematically studies the processes of transmission of knowledge in the broadest sense – covering science and cultural practices – that are organized by institutions specialized for this purpose (Chevallard and Bosch, 2020; Schneuwly, 2020). The original Greek term διδάσκειν [didáskein] mainly means “to teach, to be a teacher, to train,” as well as “to learn by being taught” and “to put someone in the position of learning” (Schneuwly, 2021). Didactics in the various disciplines deals with the relationship between three components: knowledge (including skills), teachers, and pupils. Here, I adapt this ternary approach to the components song, teacher, and pupils, drawing on my research on children’s song acquisition and development (e.g., Stadler Elmer, 2002) and on a research project on song teaching carried out by me and my team.^1^ With reference to empirical research on song teaching and learning, this paper aims, first, to render visible and explicit some of the dynamics in the process of song transmission; second, to provide a conceptual framework that allows researchers to describe song transmission systematically and explain its key aspects; and third, to render explicit some of the rules and norms that govern the practice of song transmission.

The new methods we have developed for our empirical research are descriptive in nature and serve to analyze and describe various dynamic aspects of formal song transmission. Readers who miss the application of standard methods or inferential statistics are reminded that at least since Kuhn (1962) and Feyerabend (1975), it has been considered one-sided and inadequate to regard the application of scientific methods as sufficient for the proof of scientificity. Kuhn’s and Feyerabend’s positions have been further developed in the theory of systematicity by Hoyningen-Huene (2013), which provides an important orientation for the research presented here. The philosopher of science Hoyningen-Huene answers the question about the nature of science as follows: “Scientific knowledge differs from other kinds of knowledge, in particular from everyday knowledge, primarily by being more systematic.” (Hoyningen-Huene, 2013, p. 14).

Systematicity is gradual and manifests itself in nine dimensions (Hoyningen-Huene, 2013). I articulate eight of them directly or indirectly below: descriptions, explanations, the defense of knowledge claims, critical discourse, epistemic connectedness, an ideal of completeness, knowledge generation, and the representation of knowledge. Not relevant to the present context is the dimension of prediction. In this theoretical article, the theory of systematicity is useful, firstly, to correct the misconception of science as constituted by methods or even statistics, as already postulated by Kuhn and Feyerabend. Secondly, the theory of systematicity offers a rationale for the scientification of the cultural practice of formal song transmission. Accordingly, the aims of this article can be summarized as describing and explaining the practice of formal song transmission in its dynamics and norms. By presenting a conceptual system and methodology that I apply to two empirical examples, I claim to proceed more systematically than has been the case so far in this research field.

### Intergenerational Song Transmission as a Cultural Practice

Before I concentrate on formal song transmission, it is important to consider this cultural practice in a broader context. The overarching concept is *intergenerational transmission*, without which there would be no human culture at all. In informal and formal cultural practices, the leader performs and guides selected pieces of a memorized repertoire, drawing on an ample supply based on traditions extending back over centuries and, ultimately, even millennia of acquired cultural lore transmitted down the generations (Merker, 2012; Morley, 2013). Following Merker et al. (2015), the process of cultural evolution is gradual, unconscious, obligatory, “and restructures the cultural corpus in ways that increase its salience, expressive economy, communicative generality, and grammatical power, all of which turn on enhanced communicability and learnability in various ways” (p. 2). The present-day practice of song transmission is based on biological and evolutionary adaptations. Hence, each song performed in situ for and with children represents a cultural practice that functions on the basis of biological constraints and according to norms and rules that are mostly unconscious and implicit.

In both ontogeny and cultural history, song transmission was practiced informally before the institutionalized form and thus precedes it. The institutional form serves the purposes of organized and deliberate cultural transmission and presupposes a society that has a differentiated division of labor, whereas informal transmission is much less regulated. The latter is typically performed with infants and young children in familiar settings, dyads, and small groups. It may also occur among peers or as parts of religious or ritual practices. At early stages, parents and caretakers intuitively use musical features in their child-directed vocal communication. They do this in order to establish and maintain states of joint attention and shared intentionality, as well as to prepare common ground for building up communication and emotional bonds with the youngsters (Papoušek and Papoušek, 1981). Early vocal communication by both participants is considered the manifestation of biological predispositions of support for infants’ vulnerable regulation of their physiological states (Papoušek and Papoušek, 1987). These early processes of adaptation and acculturation are viewed as affect-regulating support for building social bonds. Infants’ vocal play and its counterpart, caretakers’ musical features in intuitive communication, including children’s songs, mark the beginnings of protoconversation, as well as the precursors of the elementary cultural practice of joint singing. The youngest are introduced into the surrounding culture with its conventions and the means by which to regulate affective states by mutual responsiveness. The initial practice of song transmission happens in intimate, one-to-one relationships, and its prevailing nature is playful and joyful; thus, it is affect-regulating yet, nonetheless, rule-governed.

Many nations organize their education system by requiring children to attend school, where they are taught by teachers. Formal education typically begins when children enter kindergarten or elementary school. Here, educators and teachers tacitly presume that all children have been introduced into the song culture prior to attending school and that they are able to decode what is expected during music lessons in class. Song transmission in educational institutions is formal because a specialized person – a teacher – is mandated to transmit cultural rules through a song repertoire to a younger generation – the pupils.

### Cultural Practice and Normativity

By denoting song transmission as a *cultural practice* – be it informal or formal – I rely on the practice idiom that is widely used in social sciences and philosophy (e.g., Brandom, 1994; Turner, 1994; Schatzki, 1996; Rouse, 2007). For Turner, practice is variously interchangeable with tradition, tacit knowledge, paradigm, presupposition, and much more, whereas Rouse emphasizes norms as a constituent of practices:

Practices are constituted as such by the mutual normative accountability of their performances to “norms” that are always at issue within the practice. To say these norms are “at issue” is to say not merely that the norms change over time. It indicates that change results in significant part from the ongoing effort to sustain a common practice accountable to norms, even though what the norms are is not yet settled (Rouse, 2015, p. 152).

The widely discussed notion of “norm” is understood to concern what “ought to be” (Broome, 2018), that is, values and judgments of what is “correct,” “appropriate,” and “accurate” and their respective negations. Although joint singing belongs to cultural traditions, it has not yet been considered from the perspective of a normative practice. By doing so here, I argue that joint singing or song transmission is a prototypical cultural practice and, specifically, that it is multilayered in its normativity for at least three reasons. First, its content – songs – is deeply rooted in a culture’s traditions, which bear a kind of authority regarding adherence to norms and rules. Second, the actors involved in this didactic practice have defined roles: a responsible teacher guides pupils, who are expected to be motivated to follow. The overarching goals of song transmission are tacitly and collectively agreed upon, and when institutionally regulated, they have some national legal basis. Third, the teaching and learning of songs require complex in situ actions whose norms and rules are enacted very quickly and, thus, mostly implicitly. However, the agents’ involved in song teaching and learning do not need explicit knowledge of the rules that govern their actions to achieve the goal of transmission. Nevertheless, to understand this cultural practice from a scientific point of view, it is important to articulate and explicate the inherently enacted norms and rules.

The concept of *normativity* implies the absence of a true or untrue basis: actions and action-related statements are not verifiable or falsifiable but rather valid or invalid, correct or incorrect, and appropriate or inappropriate. Due to this lack of verifiability of the truth, validity must be negotiated, for instance, on the basis of traditions, established conventions that are changeable, or authority. In order to clarify further aspects of normativity for the present context, I adopt the three main types of norms introduced by the philosopher von Wright (1963) – prescriptions, customs, and rules –, and use them to scrutinize the practice of song transmission and to reconstruct some of its normative layers.

The first main type, the state laws in terms of legal *prescriptions* (only briefly mentioned here), articulate the political and educational interests of a society or nation in the issue at stake. For instance, certain practices can be prescribed, banned, or censored. Formal song transmission happens in institutions and is most often part of school curricula. This type of norms – prescriptions – focuses on the embedding of song transmission within a larger context. First and foremost, it includes historical aspects. Traditions may be the most powerful underpinning influence in maintaining rituals, including rules and norms. Second, at the national level, the representation and maintenance of a nation’s identity and heritage is of interest; hence, at regional levels, educational institutions are commissioned to implement curricula. Third, schoolteachers are mandated and, thus, obliged to follow the curriculum. For them, being employed and accepting an obligation implicates judging, evaluating, and monitoring their own work, along with accounting to others for the fulfillment of their obligations, including the transmission of songs. Obligations are subject to self-evaluation and, at some points, to professional judgment and evaluation. Thus, teachers are guided by regulations, norms, and rules that frame their pedagogical interactions with children. Specifically, the teaching of songs is expected to adhere to preset criteria, such as children’s learning outcomes in the form of correct performance and familiarity with the prescribed repertoire. To fulfill their duty, teachers take for granted children’s motivation, their capacity to learn, and their cultural learning experiences prior to entering school. The influence of early individual experiences is difficult to assess and has often been simplistically diagnosed in terms of talent or giftedness or the lack thereof. Hence, teachers have to cope with and adapt to heterogenous conditions. However, depending on their personal norms or standards regarding their duties and on the institutions’ policies governing the implementation and monitoring of quality, teaching songs may at times be considered important or unimportant. As a consequence, the appreciation of this practice varies, ranging from high to low or from desirable to neglected to ignored. What happens in a class is thus not up to any individual’s free choice but is influenced by values and norms represented and enacted on national, community, and institutional levels and by professional training and political conditions. In saying so, I emphasize an essential property of norms: the issue at stake with norms is not about truth and facts, as it is in the human and natural sciences, but about negotiations of values and, as a consequence, power relations. Roth (2016, p. 46) states that there is no escape from the paradox that “any application of norms already presupposes and employs a special capacity for normative judgments.”

The second main type of norms proposed by von Wright (1963) is *customs* which give directives and tell how actions are carried out and what the corresponding social practice looks like. The notion of ritual culture, as conceptualized by Merker (2005, 2009), fits into this type well. In essence, it is about obligatory adherence to arbitrary forms; the prototypical cases are systems with long traditions, such as language or music systems. While languages are the richest, most important, and most powerful symbolic systems, music belong to the domain of rituals. Ritual actions, which are typically characterized by repetition and variation, can serve interindividual, behavioral, and affective synchronization, as they enable the anticipation of upcoming events (Merker, 2009). Ritual performances further involve the correct performance of a procedure or appropriate rule-following in order to achieve an aesthetic form and respective feelings. Hence, this type of norm is pertinent to song leading – a technical term for conducting or leading a group in song.

The third main type of norm proposed by von Wright (1963) is *rules*, such as those that prototypically govern games like chess but also those that take on a more flexible form as grammars. I will take up this conception in section “The Musico-Linguistic Rules: The Grammar of Songs for Children”, when I introduce a grammar of songs for children. There is a consensus to conceive of norms as generic and broad notions and rules as types of norms that are made explicit (see e.g., Brandom, 1994).

After framing song transmission as a cultural practice governed by rules and norms, next, in section “Research on Formal Song Transmission and Implicit Norms”, I report on recent research in this domain with a focus on how normativity is handled. The pivotal study by Liao and Campbell (2014, 2016) serves as an example. It is situated at kindergarten level, which is one of the first institutions children adapt to and in which they formally begin acquiring cultural lore, including songs. Although this study appears to be the first to explore song transmission and seems preeminent in providing a detailed analysis of it, the norms and rules in question remain largely implicit. This raises questions and highlights the need to address normativity.

In this paper, I argue that a structural and didactic conception of song transmission contributes to rendering unspoken normativity explicit. Therefore, in section “The Musico-Linguistic Rules: The Grammar of Songs for Children”, I make explicit the rules that govern children’s songs as an elementary, traditionally transmitted cultural device. I propose a slightly revised version of the grammar I published in the German language (Stadler Elmer, 2015) and only partly in English (Stadler Elmer, 2020). In section “Methodologies and Examples of Research on Teachers’ and Children’s Song Constructions”, I present two empirical examples, one from my own research and the other from my team’s current research project, in order to demonstrate a structural and didactic approach. The first shows a yet unpublished excerpt from a child acquiring a song from a study partly published in 2002, and the second concerns a current study on teachers’ song leading in class. One of the characteristics of a structural approach is using concepts and methods that permit the units and their interrelations involved in the transmission process to be maintained, (i.e., the song, teacher, and pupils). Both cases make evident that the structural analysis of agents enacting songs requires a conceptualization of the normative layers and rules that govern the teaching and learning. I argue that explicating the different rules and norms helps to reveal and illuminate the inherent normativity of the formal song-transmission practice. Finally, in section “Conclusion”, I outline a synthesis and some advantages of using a paradigm that seeks to describe and explain formal song transmission as a ternary didactic system rather than studying this process by focusing selected features such as pitch accuracy.

## Research on Formal Song Transmission and Implicit Norms

Cultural practices are typically enacted on the basis of implicit norms and rules that may, therefore, remain largely unconscious. In didactic research, however, norms and rules are of central importance because they govern teaching and learning in relation to subject matter. Within this normative field, the explication of norms has a crucial bearing on how actions are judged, evaluated, or justified. Because of the social nature of singing as a cultural practice, the norms and rules are taken for granted and often remain unquestioned and poorly reflected in the research literature. In the following, I will elaborate on this assertion with a focus on formal music teaching at school.

### Research on Formal Teaching of Music and Song at Elementary Level

With regard to research on the teachers’ role, two major topics prevail: one consists in giving advice and instruction on how to teach songs to young children, and the other reports shortcomings in practice and expresses the need for improvements. Research on generalist teachers’ song leading reports this practice to be notoriously inadequate, with teachers ill-prepared and lacking musical skills and knowledge. Specifically, many researchers have identified that generalist teachers lack the confidence and necessary musical skills to transmit songs to children. They hold the opinion that generalist teachers teach songs inadequately or do not teach them at all (see e.g., Hennessy, 2017; Swain and Bodkin-Allen, 2017). There is a consensus that generalist teachers should develop competences to mediate children’s musical learning and should build their confidence in doing so. After working with generalist teachers for more than 30 years, Hennessy (2017) concludes that they can and should develop and strengthen the following abilities in the process of training: maintaining a steady pulse, clapping, speaking or moving rhythmically, using their voice expressively whether singing or speaking, and recognizing simple patterns, forms, and textural features when listening (p. 3).

I argue that didactic research on formal song teaching can make a promising contribution to more systematized norms, as this practice represents a prototype of such a process. However, there is still a long way to go before we can conceptualize a firm foundation of the dynamics between teacher, knowledge, and pupils and render their multilayered normativity more explicit.

### Observational Research on Song Leading

As a next step, I report on the only observational study that we – as a research team – have found so far on song leading. This study contains many aspects of tacit and implicit evaluation bearing on normativity. In their pioneering work, Liao and Campbell (2014, 2016) aimed at comparing differences in approaches to teaching children’s songs by kindergarten teachers in Taiwan and the United States. They pursued three goals: (1) to examine components of the song leading process practiced by kindergarten teachers, (2) to examine teachers’ starting pitch in singing children’s songs, and (3) to compare teachers’ song leading in Taiwan and the United States.

Five public school kindergarten teachers in Taipei, Taiwan, and five in Seattle, United States, participated voluntarily in this study. They were asked to teach six children’s songs on six different occasions. Non-participant observations (video recordings) were undertaken to identify the processes by which these kindergarten teachers taught songs. Interviews and field notes provided further data. Each teacher was observed during her singing activities with children in twenty- to thirty-minute sessions. According to Liao and Campbell (2014, 2016), most kindergarten teachers were proficient in applying materials and resources in teaching the songs.

A particular focus of Liao and Campbell was the *starting pitch* of the teachers’ singing. They accessed “accuracy of pitch” by identifying the initial sung sound with the help of a piano, without discussing the reliability and validity of this method. They reported that the teachers had the capacity to provide a starting pitch for the children’s voices, but that in general, the teachers sang too low. The authors concluded that most of the kindergarten teachers did not provide appropriate vocal models and did not clearly employ a song leading process. Most teachers did not provide a reference note or starting pitch, with the result “that their children joined the singing several pitches into the first phrase of the song or drifted in at a later point along the way” (p. 157). Most gave little feedback to children on the accuracy and quality of their singing; however, most teachers “set a pulse, provided a sense of meter (however inaccurate) and cued the children’s singing with a gestural signal of some sort” (p. 157).

On the one hand, this is a groundbreaking study because it analyzes and describes various and detailed aspects of song leading. On the other hand, the researchers approach the song leading process using certain traditional concepts from the pedagogical literature. In doing so, they fall into the trap of adopting unspoken and selected norms. For instance, the focus on the accuracy of the starting pitch appears as a single and isolated criterion without an explicitly stated rationale. I will return to this issue by claiming that a grammar of children’s songs defines structural relations of the song as a unit, which allows single features or elements to be treated and justified as integral parts. Another implicit normative account the authors use to describe and evaluate song leading practices concerns their naming many inadequacies and shortcomings. Here, they join the tradition of considering generalist teachers as musical dilettantes and of expressing unspoken demands on them. Indeed, as will be shown later, the process of teaching a song in class is a complex endeavor. It is insufficient to use certain selected criteria and implicit norms for its description, analysis, and evaluation because this often leads to unjustified judgments about generalist teachers’ aesthetic and pedagogical work and to the neglect of their intentions. Against this backdrop, I argue that the formal practice of song transmission calls for a theoretical foundation that aims to explicate the norms and rules used in order to describe the didactic processes that take place between teachers and learners; moreover, it needs to account for the actors’ and researchers’ views to understand and negotiate their judgments and evaluations.

## The Musico-Linguistic Rules: The Grammar of Songs for Children

In this section, I proceed to make explicit the norms and rules that govern the formal transmission of songs. The songs that introduce children to the musico-linguistic rules of their environment can be considered an elementary and rule-governed form or genre. Because of their introductory or elementary nature, I refer to this genre as “songs for children” and also as “children’s songs,” although these songs are not created by children but usually dedicated to them. The song – and the rules or underlying grammar – is the teaching and learning object in the didactic system. My attempt to explicate the rules of this elementary genre and formalize a grammar involves reference to general and abstract features, as well as to technical terms from various disciplines [music, linguistics (phonology, morphology, etc.), acoustics] that have been empirically generalized and are used worldwide to communicate about musics, languages, and songs. This allows basing conceptual analysis on established scientific knowledge and the application of technical terms to the analysis and description of the practice of song transmission. This scientific strategy can be seen as rooted in the dimension of “epistemic connectedness” of Hoyningen-Huene’s systematicity theory (Hoyningen-Huene, 2013). The Children’s Song Grammar presented below thus draws on existing scientific concepts. Whether and how these scientific concepts are also used in other cultures outside the European languages I know is beyond the scope of this article.

### Attempts to Formalize Melodies

What are the essential properties and the rules of children’s songs as the introductory genre for children? The music ethnologist Nettl (2000) describes the features of simple, universal children’s songs as consisting of short phrases, repeated with small variations, and covering three or four different pitch categories in the range of a fifth. Yet, children’s songs are not only melodies but also always a combination of two different yet intertwined systems: music and language. Although there is much research on the comparison of music and language (e.g., Patel, 2008; Rebuschat et al., 2012), only rarely is the combination of the two addressed with respect to the traditional form of songs (Stadler Elmer, 2002).

The rules of music systems are a key issue, and composers and researchers have long been proposing various attempts to formalize them. Of primary interest here are propositions for analyzing and describing performances, with the aim of helping to unveil implicit rule-following. Related, but secondary, is the act of transforming a notation system into a performance, where it serves as a model and memory aid. Regarding the detection of rule-following, Sundberg and Lindblom (1976) for instance, described Swedish nursery tunes in terms of a generative rule system. Langer (1953) mentioned that music, like language, has discrete parts that can be combined in a variety of ways to make new expressive wholes. Lerdahl and Jackendoff (1983) published their generative theory of tonal music, and Povel (2010) designed algorithms for generating melodies. All these approaches focus on melodies as hierarchically organized units and deal with tonality and with musical time in terms of metrically based durations with proportional values. However, and this is most remarkable, these attempts have not dealt with the combination of melodies with texts. This combination is the main topic of the next section.

### Elaboration of a Grammar of Songs for Children

Because there has been no systematic attempt to formalize the rules and principles of children’s songs to date, I have designed a grammar for this genre (Stadler Elmer, 2015, 2020). The aim is to explicate the abstract rules of how the linguistic and musical elements are combined into a coherent unit: a song. Before I go into sketching out this grammar, it is necessary to show how music and language – with the focus on song and speech – are constructed as generative systems.

In 2002, Merker proposed that human music, like language, is a generative system. He elaborated the core principles of music as an open-ended self-diversifying (Abler, 1989) or generative system: humans do not exploit the full *continua* of frequency (pitch) – of their voice or of instruments – and time; instead, they create limited sets of discrete categories and combine them into complex patterns. A generative system combines *discrete elements* (“particulates” that do not blend to an average upon combination) in composite patterns of boundless variety. In music, the particulate elements are obtained by discretizing the continua of pitch and time, yielding tones of distinct pitches to which a melody can return (such as the seven tones of a diatonic scale) and distinct durations (Merker, 2015). When these durations are related by whole integer proportions and combined in cycles and stress patterns, they supply the elements for the rhythms of all rhythmic (metric) music.

The idea that language and music are generative systems goes mainly back to von Humboldt (1836, p. 70). He characterized language as a system that makes infinite use of finite media, of which the synthesis creates something that is not present per se in any of the constituents. In languages, the discretized elements are the phonemes. Each language uses a select sample of the phonatory capacities of the human vocal apparatus. These elements are combined into syllables and words, and these, in turn, into sentences in boundless variety. Specific to each language, these combinatory rules govern word formation on the basis of metrically accentuating the syllables. An accent or stress is a *relational* feature of a sung or spoken syllable defined by the contrast to its adjacent syllables in terms of contrasting intensity, duration, pitch, or a combination of these (Hall, 2000; Ewen and van der Hulst, 2001; van der Hulst, 2014). In other words, accent, stress, or strong and weak beats do not have a unique phonetic, musical, or acoustic property; rather, they exist only in contrast to adjacent syllables with respect to intensity, duration, or pitch and, thus, are *relational*. Cross-linguistically and musically, it is highly probable for stressed or accented syllables or sounds to exhibit greater loudness, longer duration, and higher pitch levels than unstressed syllables (e.g., Kager, 2007; Santa, 2019). Whereas intensity, duration, and pitch are continuous and can be measured or calculated, and furthermore, pitch and time are discretized in music (see aforementioned, Merker, 2002), stress or accent is relational in the manner explained above (see also Table 1 below).

**Table 1:** Syllables and their properties are differently organized in song, reciting poems, and in speech.

	Continuous	Discrete categories	Relational	Rules	Organizational units
Song	Intensity	Pitch, duration	Accents: musical and verse meter	Tonal, timing, lyric	Syllable, phrase
Reciting/chant	Intensity, pitch	Duration, phonemes (with onomatopoetic features)	Accents: verse meter	Timing, phonemes (rhymes)	Word, verse line
Speech	Intensity, pitch	Phonemes	Language-specific stress patterns	Prosody, morphology, syntax	Word, sentence

### Identification of the Building Blocks

The starting point for designing a grammar that combines the musical and linguistic systems in an elementary manner as in songs for children is the assumption that syllables are an element common to both. Syllables, consisting of vowels (V) and combined with consonants (C; VC, CV, CVC), entail both musical and linguistic properties. Researchers have shown that the repetition of fragments of spoken language is soon perceived as singing (see e.g., Deutsch et al., 2011; Tierney et al., 2018). The reasons for the dual characteristics of syllables lies, first, in the fact that the sonority of a syllable, when its duration is prolonged, creates the impression of singing due to pitch becoming salient and modular. This statement can be specified in four ways:

- Sonority is a technical term in phonology (e.g., Goldsmith, 1990; van der Hulst, 2014). High sonority tends to align with the head of a stress foot (e.g., de Lacy, 2006).
- Historically, many researchers have cited, the feature of vowel duration to distinguish speech from song (e.g., Stumpf, 1911; Langer, 1948; Dowling, 1984; Stadler Elmer, 2002).
- Acoustically, the effect of the lengthened and shortened duration of vowels on the auditory perception of song and speech can be easily visualized and verified with digital tools.
- In the literature, the temporal correspondence of short vowels with speaking and of long vowels with singing has been visualized by, for example, Lindblom and Sundberg (2007, p. 697, Figure 16:38).

Second, when repeating syllables and varying inherent features of intensity, sonority, duration, pitch, another feature then emerges: a stress pattern or periodic accentuation. These inherent constituents of syllables and the properties vocally produced by repetition and variation are fundamental to the organization of song and speech. As explained above, stress or accent is a *relational* property that, when produced periodically, is called musical meter in melody and verse meter in poetry, and in song, the stress patterns of melody and lyrics come together.

In each of the two vocal modes, and also when reciting poems, syllables are the building blocks, although in each case, they are differently organized. Poetic language occupies a position in between song and speech by exhibiting features of each. Table 1 presents an overview of how syllables, as a common basic unit, are organized differently in singing, reciting, and speaking due to different generative principles and combination rules applied to them.

The metrical rules of song, recitation, and speech differ. For speech, accents primarily govern word formation, and the segmental syllables meld together while talking. For song, meter governs the synchronization of the melody with the lyrics by means of the periodic accentuation of syllables, and the sung syllables meld into a melody while retaining their segmental character through their metrical structure. In reciting poems, the syllables are bound to metric patterns (binary: trochee, iambus; ternary: dactyl, and anapest), and onomatopoetic features begin or end verse lines as alliterations or cross rhymes. Recited poetry is bound to rhythmic and rhyming patterns although, in contrast to song, without melody.

### The Notion of a Grammar

A grammar should define at least the building blocks and syntactic rules that permit explication of how typical children’s songs are constructed and recognized as exemplars of this genre, be they sung, technically reproduced, or symbolically represented. By knowing the constitutive elements and rules, it is possible to describe or even to predict the process of how a target model is transformed into adopted versions and how this process is monitored and evaluated by comparing the various song versions. Just as the grammar of a natural language makes it possible to produce an utterance, monitor it, and evaluate it as either correct or incorrect or well-formed according to the rules, the Grammar of Songs for Children serves to identify a song as similar to a target model, to assign it to the genre “children’s songs” or not, and to describe a sung song as more or less well-formed according to the rules. The term well-formedness has become established in linguistics and also art theory (Plümacher, 1999) to refer to a more appropriate kind of correctness that corresponds to the character of a grammar.

In children’s and folk songs, the lyrics follow poetic language rules and thereby provide not only a periodic meter that needs to be combined with melodic meter but also rhymes that mark the ending of a verse line or phrase. It follows that the combination of lyrics and melody – a defining property of children’s songs – may create tensions between competing syllable accentuations, namely, linguistic stress patterns for word formation, poetic verse meter, and melodic meter. What is required here are combinatory rules that constrain the possibilities. After having defined the syllables as the building blocks of children’s songs, the next step in constructing a Grammar of Children’s Songs consists in identifying the syntactic rules.

The Children’s Song Grammar presented here grew out of my research and analysis of countless songs sung by children, as well as song models and the necessity of explicating the inherent structures. Inevitably, normative questions arose about what is considered correct or incorrect and for what reasons. The goal of this Grammar of Songs for Children is to identify and explicate the recurring elements, patterns, and regularities that underlie both the song as an abstract and rule-governed model and the act of singing, that is, using the voice to produce sound patterns that conform to some minimal cultural rules. The model song typically represents a general and abstract device collectively shared, and the act of singing – unless recorded – is the epitome of a transient event, although densely structured and resembling the general model.

### The Grammar of Songs for Children

**Figure 1:**
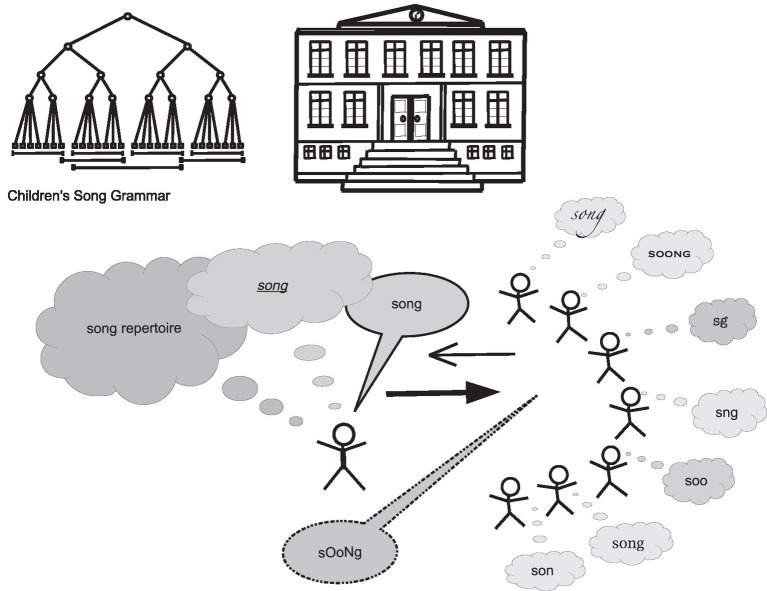
A schematized representation of formal song transmission. A teacher teaches a song to school children. Finally, the acquired song should have a structural similarity to the model.

In order to reconstruct formal song transmission as a practice, it is necessary to make explicit the rules that govern songs as vocal productions and as abstract models in written from. The completion or effectiveness of song transmission can be evidenced by similarity between an original or target song and the adopted versions. The expected result is an identical or at least highly similar form. Ideally, teachers achieve this goal by constantly monitoring and evaluating the process. They know the model and pass it on by intuitive or deliberate instructions that aim to make the target attractive and progressively adoptable. The learner is assumed to have the vocal capacity to grasp and acquire the rules of the target song, to memorize it, and to feel the effects created by the sharing of synchronized and synphonized^2^ actions and repetitions. Figure 1 schematizes the process of song transmission. Characteristic of this process is that songs as vocal products are the epitome of an ephemeral event. Whereas the teaching and learning of a new song occurs as a very fast interaction, the researcher must decouple this process from its actual temporality. To achieve this, an important assumption is to consider each song as an exemplar of the repertoire of the genre of children’s songs. This repertoire shares essential features with all other instances of this group, which is defined by these features. Children’s songs are used as an elementary practice to introduce the next generation into the musico-linguistic culture. Hence, the features must have a basic character, along with rules that govern the configuration of the constitutive features. These considerations have guided the process of making explicit the Grammar of Songs for Children presented here.

#### The Grammar of Songs for Children: General Properties^3^

1. Songs for children consist of lyrics (a verse or a poem) and a melody.
2. As the two *generative systems* – music and language – are both involved, an infinite variety of songs can be potentially generated.
3. The building block of both systems of song-making is the *syllable*, consisting of a vowel (V), alone or combined with one or more consonants (C). Syllables inherently have linguistic and musically properties: V and VC with pitch, duration, and accents.
4. Songs are *hierarchically organized*.
5. Lyrics and melody are relatively autonomous (see 2), thus forming parallel hierarchies (see 4). Ideally, lyrics and melody are well matched. If this is not possible, one or the other is subordinated.
6. The verse meter of the lyrics and the musical meter of the melody simultaneously govern the timing of the syllables. This may create tension and call for compromises.
7. Symmetries of temporal and sonorous forms aim to create a *well-formed whole* or Gestalt.

#### The Grammar of Songs for Children: Timing Rules^4^

1. The syllables are timed by a regular pulse and by periodic accents that yield a binary or ternary meter. Thus, basically, the duration of every syllable is equal (syllable-timed).
2. The duration between two isochronous pulsations (onset time of syllables) is the basic temporal unit and determines the tempo. In songs for children, the tempo is regular and constant, and usually does not change.
3. In songs for children, pauses appear at the end of a phrase and not within.
4. All the durations of the syllables and pauses are defined in relation to the pulsation. Syllables and pauses can be long or short, and the longer duration is always two times the lengths of the basic unit, the pulsation, and the shorter is half of it. Usually in songs for children, these three duration ratios are used, that is, the basic unit of the pulsation with double or half values.
5. Once the binary or ternary meter (measure – a pattern of periodic accents) is set, it is valid for the entire song. Songs for children do not change their measure or meter.
6. The entire duration of a song is segmented into phrases (groupings) of equal duration. As the subunits, the phrases can be repeated. A phrase normally consists of two or four measures and rarely of only one measure.
7. The duration of a phrase is related to the duration of exhalation. Breathing occurs between phrases. Phrases are shaped by the melody and the lyrics: they may consist of a short and easy-to-memorize melodic motive and, simultaneously, of a verse line.
8. Songs for children have an even number of measures, typically four, eight, ten, twelve, fourteen, or sixteen.
9. A song and also the phrases may begin with an incomplete measure on an upbeat. The upbeat is always unstressed, for example, *Incy Wincey, Spider; Did You Ever See a Lassie?;* or *The Sandman Comes*. As a consequence, the final measure is reduced by the duration of the upbeat, which together yield the duration of a full measure, thereby maintaining the continuous metrical timing of the lyrics.
10. The temporal structure consisting of meter (measures) and phrases is the framework within which the syllables (including tones) are organized. This means that no syllables or tones exist outside the defined meter, the even-numbered quantity of measures, or the phrases. All syllables and tones are integrated into the meter of the melody and of the lyrics.

#### The Grammar of Songs for Children: Tonal Rules

1. Songs for children are nearly always in major keys and rarely in pentatonic scales or minor keys.
2. The major key, once chosen, is maintained throughout the song. There is no change of key.
3. Songs for children begin with one of the three tones of a tonic triad (do, mi, sol); thus, the key of songs for children is marked in the first measure.
4. The melody consists of a limited set of tones of a major (or minor) scale (with seven tones: do, re, mi, fa, so, la, and ti). Again, as a consequence, the key does not change (cf. tonal rule 2).
5. The pitch range of the melody does not exceed a major ninth (fourteen semitones, or an octave and two semitones).
6. Usually, songs for children end on the root of the key (do), most often as a descending melody. So-called “chain songs”^5^ may end on the second tone of a tonic triad (mi) as do, for instance, *In Mutters Stübele* or *Laurenzia*.
7. Each tone is connected to a syllable. Therefore, the number of syllables and the number of tones are equal. Only rarely is it the case that a syllable is combined with two melodic tones (e.g., in *Bye*, *Baby Bunting*).
8. If the melody moves with an interval in one direction (a fourth or larger up- or downward), the subsequent movement changes direction.

#### The Grammar of Songs for Children: Poetic Rules

1. The lyrics follow the rules of poetic language. This means they have a meter and verse lines that end with rhymes (pair and cross rhymes).
2. The verse meter defines the periodic stress pattern of the syllables (or words). The binary patterns are trochee and iambic, and the ternary patterns are dactyl and anapest.
3. The verse meter matches the musical meter (measure, binary and ternary meters) of the melody to yield a well-formed entity or Gestalt. This means that the stressed syllables of a word correspond exactly with the stressed beats (accents) of the musical meter. Text and melody provide a temporal framework in which the lyrics and the melody are simultaneously synchronized. Their relative autonomy (cf. general properties 5) makes it possible to match a single melody with many verses.
4. Well-formed synchrony of melody and text can be achieved by subordination, for example, by adding or omitting syllables (abbreviating a word) or tones, as when fitting names into birthday songs and greeting songs.
5. The verse lines correspond to the phrases in that the number of syllables equals the number of tones (one-to-one relationship) and by ending on pair or cross rhymes, thus interconnecting the verse lines and the phrases.
6. The simplest lyrics combined with a melody consist of one or a few repetitive syllables. This pattern is characteristic of the refrain of a verse. Repetitive syllables are also sung in order to replace or extend the lyrics for many reasons, for example, well-formedness, completeness, simplicity.
7. Unsemantized, repetitive syllables – for the sake of their sound – are typical elements of lyrics in songs for children.

#### A Prototypical Form of Songs for Children

As song for children may have the following properties:

- a narrow pitch range (fifth, octave)
- repetitions of the same pitch
- small melodic intervals
- all tones belong to the same key
- tones have two or, at most, three duration ratios, determined by the pulsation
- each syllable corresponds to one tone
- eight measures, or equally timed phrases yielding a symmetrical structure
- repetition of phrases

**Figure 2:**
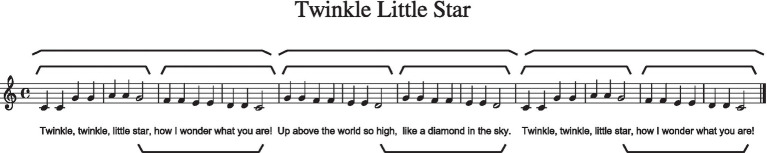
A typical children’s song that follows the rules of the grammar. Above the notes, the phrases and subphrases are marked by brackets, and the ones below the text mark the rhymes. Note, for instance, the symmetrical organization and the salient boundaries of the phrases and subphrases.

Figure 2 represents the well-known children’s song *Twinkle Little Star*. It follows all the rules and has the properties of a prototypical exemplar.

#### Functions of the Grammar of Songs for Children

By analogy with the role of grammar in linguistics, the Grammar of Songs for Children presented here serves to organize parts or elements systematically into a coherent unit, be it a sentence or a song. In the present context of research on song transmission, I point out three interrelated functions of the Grammar of Songs for Children:

1. It is a tool to reflect songs at an abstract level and thereby make them an object of conscious consideration and communication. When we are able to analyze and conceptualize the elements and rules, they gain a new status and become objects of reflection with some general validity. A grammar, thus, is a mental reference system for the evaluation and judgment of one’s own productions, as well as those of others. To explore and understand how people share and shape their culture, it is essential in research to know the abstract rules that guide such behavior.
2. It is a tool that provides an orientation for and a justification of aesthetic judgments and feelings. A grammar allows aesthetic judgments about whether rule-following yields coherence that is well-formed. The striving for well-formedness in vocal utterances – speaking and singing – and in other media such as writing and painting, is rooted in the Gestalt principles on the one hand (e.g., von Ehrenfels, 1890; Wertheimer, 1923) and in the ritual character of song and music (Merker, 2005, 2009) on the other hand. Both, well-formedness and Gestalt principles correspond to aesthetic dimensions with roots in human evolution (Merker, 2018) and sensuality (Valsiner, 2020). The concept of well-formedness originates in linguistics and refers to a perceived correct or coherent application of generative rules when, for example, making a sentence (Meyer, 2009), a song (Stadler Elmer, 2015), or drawing a certain type of picture (Plümacher, 1999; Stadler Elmer and Weniger, 2019). Intuitively, a sentence, a melody, or a song that conforms to grammatical rules appears well-formed. Accordingly, language and music competencies show up in implicitly grammatically oriented judgments, as well as in the formation of statements or expressions with reference to well-formedness. We acquire this grammatical knowledge from early childhood, and it manifests itself in socially shared cultural practices, usually without explicit awareness (Stadler Elmer, 2020). In general, systems consisting of defined elements and combinatorial rules – so-called generative systems – imply the property of well-formedness with respect to the products arising from them.
3. It is a tool that provides an orientation for and a justification of pedagogical judgments and decisions. Conceiving of teaching and learning songs in terms of transmitting a set of immanent rules or a grammar helps to analyze and better understand the complex practice of song by means of a normative frame of reference. Without knowledge of the cultural rules that govern vocal expression in its shaping as song, there is no structural or conceptual framework on which to base description, analysis, or theorizing. Thus, the Grammar of Children’s Songs presented here makes it possible to identify a song as an exemplar that conforms grammatically on the basis of the linguistic-musical rules applied. At the same time, the grammar can be used to determine the extent to which an exemplar deviates from it. A deviation from the simple rules can mean that the song is more complex, that it can no longer be considered a typical children’s song, or even that it belongs to a different genre. Determining the structural complexity of a song can thus clarify whether a song is appropriate for the intended target group from a linguistic-musical perspective. Since songs can be used in the classroom to pursue many and varied learning objectives, a grammatical analysis of the intended song helps to identify the easy and more difficult parts and to clarify the level of requirements. Also, a grammatical analysis brings clarity to the extent to which a children’s song is well composed – again the aesthetic dimension – or to inconsistencies present that one might wish to correct oneself. This Grammar of Children’s Songs also makes it possible to analyze the actual performance of a song with regard to its regular structure and to obtain hints regarding which aspects of the structural composition can still be improved during production. If the criterion of exact intonation is solely used in the evaluation of joint singing, the focus is on just one single, albeit demanding feature. In this case, the view of the whole seems to be lost.

## Methodologies and Examples of Research on Teachers’ and Children’s Song Constructions

Having focused on the song as an object of transmission, in this section, I use the outlined Children’s Song Grammar to empirically demonstrate the perspectives of the other two didactic components, that is, the acquisition of a new song by a pupil and, as an independent case, the teaching of a new song by a teacher in class. Both cases are paradigmatic examples of how we systematically analyze and describe the process of song acquisition and song teaching based on a conceptual framework. The two examples serve to demonstrate the development of new and tailored descriptive methodologies and the strategy of obtaining new knowledge about norms in a hitherto little-studied area through systematic empirical description.

### Didactic and Structure-Genetic Conceptualization of Song Transmission

As schematized in Figure 1, the practice of teaching and learning new songs concerns one song at a time. As stated in section “The Musico-Linguistic Rules: The Grammar of Songs for Children”, each song exemplifies general underlying rules or a grammar applied in a new song-transmission context. Teachers are expected to know the song repertoire of the institution’s textbooks and to select those they can perform and teach to the class by progressive guidance. By adopting a didactic and structural view on the process of transmitting songs, one aim is to characterize the structural similarity that ought to emerge from the teaching and learning, that is, to describe how both parties vocally construct the target object, the song, while the pupils adapt to the teacher’s model. The emergence of new vocal structures expressed by pupils and those that teachers intentionally perform for them can also be conceptualized in terms of the structure-genetic view introduced by Piaget. In this view, the focus is on the genesis of vocal structures and emergent cultural forms, feelings, and consciousness in human development (Stadler Elmer, 2002, 2015). Thus, this perspective conforms to research in didactics, where teaching is conceptualized as transforming knowledge to facilitate the pupils’ acquisition (Dowling, 2020; Schneuwly, 2021), hence, implying the emergence of new structures.

The first empirical example draws on a quasi-experimental study on the song-acquisition process in children and concerns an as yet unpublished case as part of a larger study (Stadler Elmer, 2002). The second concerns insights from our current research on the development of the capacity of song leading. Both empirical cases articulate the ternary components of didactics – teacher, song, pupils – and modalities of their interrelations. The key issue is the transmission of knowledge and skills related to song as a formal cultural practice that is shared in order to deliberately transfer it from one party to another. In other words, the teacher’s goal is to enable the pupil to achieve a structural similarity between his or her vocal utterances or singing (i.e., to teach and learn a new song). How does the teacher present the song in a learnable way, and how does the pupil accomplish this task? I give the answer to these two questions below through a primarily descriptive account. However, it is based on a conceptual framework with technical terms that can be generalized to other cases in the same subject area, for example, the conceptualization of the rule-governed object as a grammar of songs for children. Therefore, the methodology and the systematic description of paradigmatic empirical examples contribute to my goal of uncovering implicit normativity and thereby increasing systematicity (Hoyningen-Huene, 2013).

### A Pupil Acquires a New Song: The Production of Structural Similarities With the Song Model

The first empirical example draws on my research on the song-acquisition process in children; thus, it focuses on pupils’ singing and less on teacher’s activities (Stadler Elmer, 2002, 2015). The quasi-experimental study concerns the above-mentioned task, that is, to teach and learn a new song in a formal setting. For this context, I composed seven new songs, of which some violate the grammar in order to assure unfamiliarity to all, and to explore children’s coping with unconventional rules. Each of the songs is connected to a picture in a book that I used while teaching the songs. More than sixty children aged between 2 and 1/2, and 9 years participated, each one in several sessions in pairs or groups of three, or exceptionally, alone with me. Here, I present an excerpt on the case of Sabine (9 years old) and her learning the song *Emma Kirsch*. For the structural analysis of her productions, I used a tailored computer-assisted acoustic method devised to analyze and graphically represent in detail the syllables and their pitches, timing, and accents (Stadler Elmer and Elmer, 2000).

**Figure 3:**
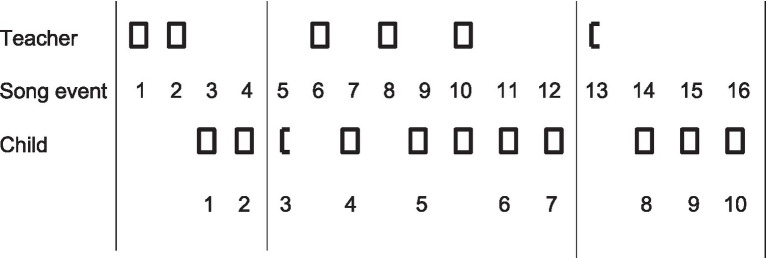
Analysis of the interaction between teacher and child (Sabine) with respect to the target song. Squares represent the song sung by the teacher or by Sabine, and half squares represent incomplete reproductions of the first part. The vertical lines separate the sessions. The interaction analysis shows the typical pattern of transmission: The teacher presents the song, and the child gradually adopts it.

Figure 3 provides an overview of the interaction between the teacher and the child, Sabine, with regard to the target song *Emma Kirsch*. It shows all sixteen events of the target song sung either by the teacher or the child. We see the expected pattern that characterizes an effective song transmission: the teacher presents a song in an adaptive way, and the child eventually adopts it. The interaction analysis in Figure 3 provides an overview of the temporal frame to contextualize all song versions sequentially produced as the model or as Sabine’s versions.

**Figure 4:**
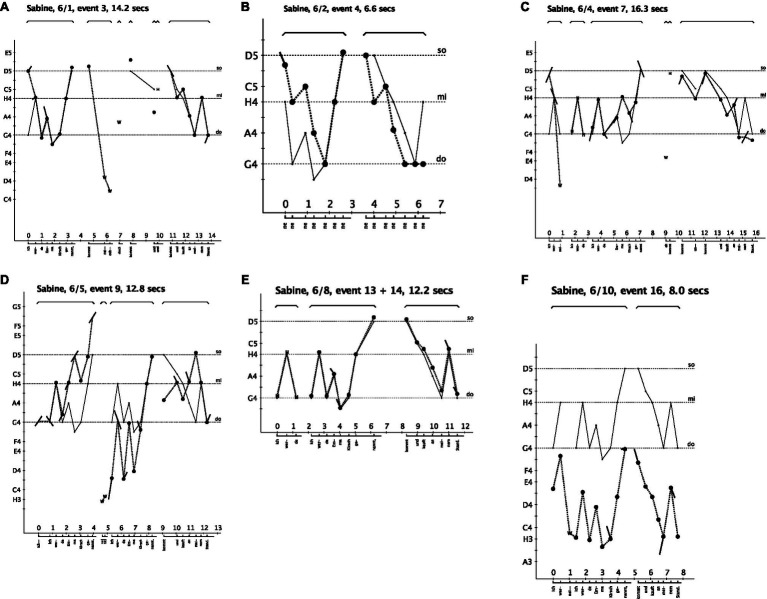
The song model is represented by the thin, continuous line. Sabine’s production is shown by the syllables and duration at the bottom and by the dots representing the quality and tonal position of the pitches which are connected by the dotted line. The x-axis represents time in seconds, and the y-axis pitch as continuum that is subdivided into the pitch categories of our Western tonal system. The symbol W means “Words,” and H stands for “Help” given by the teacher. The title at the top of each figure indicates the name of the singer, the number of the song (here number 6), the solo version, the event in the sequence (see overview in Figure 3) and the duration of the song. **(A)** Sabine interrupts her first version (see W) and is given help with one syllable (see H). **(B)** After the first reproduction, she sings a tune that she considers to be similar. **(C)** She gets help to begin the song (see H) and again at the beginning of the second phrase. **(D)** After the first phrase, she says “no, no” (see W), and she begins again. **(E)** In the new session, she asks for giving the beginning (see H). **(F)** Her last solo version (number 10, event 16), she sung rather fast and low.

How does Sabine learn this new song? Figures 4A–F show six out of her ten song versions to provide insight into her acquisition process. The figures show the results of the acoustically based analysis, and they provide a structural analysis of how Sabine – with each song version – organized her vocal expression with the intention of adapting to the model.

Her first version, represented in Figure 4A, already shows a fairly good adaptation to the model. Yet, also typical of her song inventions and learning other songs (Stadler Elmer, 2002), she interrupts her production, comments on it, pauses, and repeats fragments. Overall, I interpret these signals as indicating her monitoring the process by self-evaluation and self-judgments. Such increased self-control contrasts with the spontaneous productions of younger children. In her first version, she meets obstacles in the transition to the second phrase. Within ten seconds, she interrupts, comments, sings “kommt” with clear pitch, pauses, and sings “und” with a lower pitch, while the teacher almost simultaneously sings “und” to provide the proper pitch to continue. At second 19, Sabine begins again with the second phrase and ends the song by following the model with a minor variation. She now says: “That is a familiar tune” and produces a tune with the repetitive syllable “ne,” as shown in Figure 4B. It has the same temporal structure as the target song and, obviously, Sabine’s own phrases produced three times – the last phrase of the version in A and the two phrases in B – are very similar. They vary in conventional ways; that is, they follow the Grammar of Children’s Songs.

In the next session, Sabine begins her fourth version (Figure 4C), interrupting and getting help with the first three notes. She sings the first phrase of the target song, pauses, comments with one word, receives help with the next syllable and notes “kommt,” repeats it, and sings the second phrase by ending on the root note, and a similar pattern as produced in version two by ending three times almost on the root note as in the tune she regarded as familiar in Figure 4B.

In her fifth version (Figure 4D), she begins by repeating the first syllable, sings the first phrase by producing intervals almost fitting into the major triad upwards to the octave, but obviously too high. She says “no, no” (see W for words in Figure 4D) and starts the first phrase again, this time much lower and in a tonally unstable manner. Yet, amazingly, at the end of this phrase, she seems to have gained the tonal stability of G major again (see the patterns do – mi – so), as given in the model and as alluded to in her first phrase of this version. This song production shows that she adopts tonal patterns of the model as well as repeats her already adopted patterns; however, the combination is either not yet correct or unstable.

In the last session, in which Sabine produces version 8 to 10 (see overview in Figure 3), she wishes to be given the beginning of the song (see event 13 in Figure 3 and corresponding symbol H in Figure 4E). Thereafter, and once more amazingly, she produces the whole song while matching the model fairly well (see Figure 4E, event 14). More interesting than the pitch deviations in the second phrase is her slowing down before and during the transition from the end of the first phrase to the beginning of the second. Here, she pauses as if reflecting on the continuation.

Figure 4F shows Sabine’s final solo version, sung rather fast and low. She sung two syllables, interrupted, and started again, yet, transposed the entire tune from the original starting pitch G4 to H3.

Overall, the structural conception of songs with syllables as building blocks gave rise to an acoustically based method for the analysis of singing. The example of Sabine’s case shows this method’s capacity to provide graphic descriptions at the level of sung syllables and their timing and integration with the lyrics and the melody. Hence, the resulting Figures 4A–F show Sabine’s vocal productions and make it possible to reconstruct the process of how she elaborated song versions that are similar to the model song. One of her strategies is revealed by her second version, where she recalls a tune that she considers to be similar, and that serves as a kind of auxiliary model. It conforms well to the presented Grammar of Children’s Songs, yet seems to be a spontaneous invention by Sabine. Whether this interpretation is true or false, by song version two, she expresses a clear understanding of the general properties and tonal and timing rules of song. These internalized rules and norms guide her production and monitoring, and she is able to combine and recombine parts and construct versions that appear well-formed. For instance, most of her tunes are in G major, except for the last version, which is transposed. Overall, we observe and can say that in the end, she succeeds, although on the way, the structures of her vocalization indicate some instabilities. Her two final productions in the last session are similar to the one depicted here as her tenth version (Figure 4F); thus, it has been left out. Whereas her productions can be interpreted as exhibiting a clear intention to adapt to the given song model, the teacher likewise contributed to the transmission by presenting the song in an adaptive manner, especially by providing help prompts on demand to begin or to continue the song production.

Taken together, these insights from a case study reveal a descriptive methodology that accounts for the child’s structural adaptation to a song model that the teacher performed flexibly in adaptive ways. To achieve structural similarity between the teacher’s model and the child’s reproductions seems to be the mutually shared intention. There is no need to assume that these agents would be aware of their rule-following productions; however, as researchers interested in the norms and rules that govern cultural practice, we ought to consider these abstract levels. Making explicit some of the norms and rules means seeking what various song transmission events have in common and what could be generalizable in this context. To extract the abstract rules that govern the event is a process of systematizing. Using the concept of systematicity, Hoyningen-Huene (2013) characterizes and differentiates scientific knowledge from other kinds of knowledge, particularly from everyday knowledge such as the practice of song teaching and learning.

### Research on Formal Song Transmission: Transcription of Teachers’ Constitutive Activities

As a research team, we study formal song transmission in various ways by asking teachers to complete the task of teaching a new song of their own choice to a group of children aged between 4 and 9 years in the context of a lesson lasting between 20 and 40 min. The complexity of this task corresponds to formal cultural practice, and the task represents a prototypical and elementary form of cultural practice. We video-record the lesson, and immediately afterward, we watch it together with the teachers while interviewing them. In our research project, we use this task and procedure to study formal song transmission with two major emphases. First, we investigate longitudinally how pre-service teachers develop their professional capacity of song leading during their training by recording three lessons, each one a year apart, during their internship. Second, we study how experienced generalist teachers carry out this task. Moreover, members of the extended research group^6^ study (1) how teachers use instruments and digital media, (2) how experienced teachers teach songs in class, (3) how teachers in the teacher education institution train pre-service teachers, (4) how beginning in-service teachers cope with the transition into becoming professional song leaders in class, (5) how teachers guide the class from non-singing to singing states, and (6) how well selected children follow.

**Figure 5:**
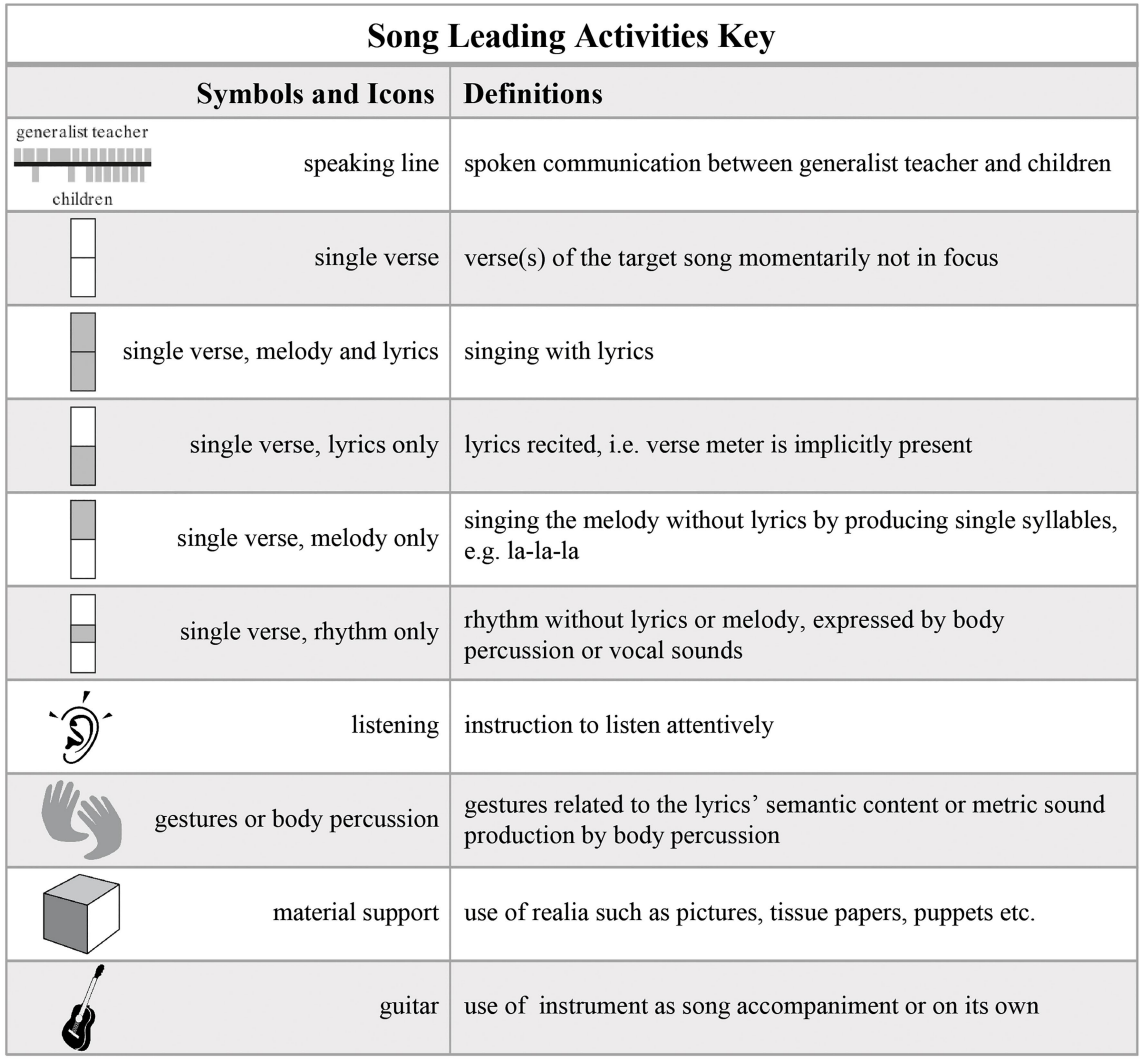
Icons and symbols that represent the recurrent and constitutive activities during formal song transmission gained at the basis of the analysis of 45 lessons. The LAMap in Figure 6 is a transcript of a lesson. Our set contains other elements as needed, such as “piano,” “percussion instruments,” and “movements” (see Savona et al., 2021).

**Figure 6:**
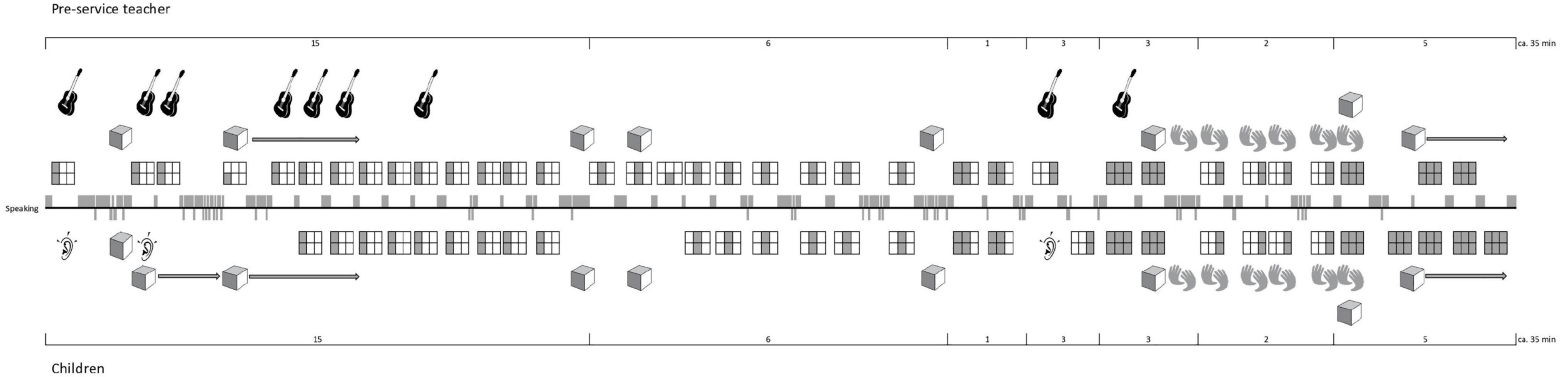
An example of a Lesson Activities Map (LAMap). It is the transcript—based on video recordings—of the recurrent activities that constitute the course of a singing lesson (see key in Figure 5). Note the visualization of the song transmission process that starts with the teacher’s presentation of the first verse, and that ends with the children’s own reproduction of all three verses (Case study with Florence, © by Savona, 2021).

Here, I only focus on the methodological step of obtaining a descriptive overview of a lesson on the basis of observation and of video recordings. This step is fundamental since song teaching and learning is an event of which the temporal and transient aspects needs to be transcribed into some kind of atemporal format in order to study its organization. Therefore, in order to transcribe the teachers’ song-related activities in a systematic manner, the project team devised a new methodology, namely, a transcription system – the Lesson Activities Map (LAMap; Savona et al., 2021) – which consists of a limited set of icons and symbols. We developed this transcription methodology on the basis of the analysis of 45 lessons. We identified the recurrent song leading activities which constitute the course of the lessons. Figure 5 shows the set of icons and symbols we used for the transcription of a recorded lesson given as a LAMap in Figure 6. The full set encompasses some additional icons; however, the system is adaptable to new challenges if required to account for a phenomenon.

The LAMap is part of our research paradigm that emphasizes a structural and didactic approach; that is, to maintain the persons – teachers and pupils – and target object involved as units and to study their relationships instead of singling out certain features or factors in terms of externally produced cause-effect relations. Hence, Figure 6 shows an example of a systematic attempt to transcribe and describe how a pre-service generalist teacher guides the pupils by working with the complete song and its parts and by using props. The focus of this transcription methodology is the song and how teachers use their knowledge and skills to perform and present it to pupils to facilitate their learning. The usual way of transforming a complete song while teaching is to separate the lyrics from the melody. To keep track of the various song transformations, we symbolize them using five different rectangles. These indicate the properties defined in Figure 5: single verse, or single verse containing melody and lyrics, melody only, lyrics only, or rhythm only. These symbols can be used to study how teachers segment or transform the song into parts and the whole throughout the lesson. The line in the middle of Figure 6 represents the spoken conversation; the field above are the teacher’s activities; and the field below shows the pupils’ activities. Note that at the beginning, the pre-service teacher Florence asked the children to listen attentively and presented the song’s first verse that she herself accompanied by the guitar. Toward the end, they sing the whole song together three times, and finally, Florence asks them to sing it without her, which they do. The LAMap in Figure 6 visualizes the progression of this process and shows the teacher’s efforts to make the song learnable for the children based on parts and the whole.

Without considering the quality of the teacher’s work with the song and guidance, and without evaluating and judging the pupils’ final performance, Figure 6 shows the expected pattern of effective transmission. It is a much more detailed transcription of the interaction analysis compared to the one given in Figure 3, which is reduced to the sequence of events in terms of the teacher’s song presentations and pupil’s solo productions. In both cases, the focus is on the teacher’s handling of the song to facilitate transmission to the pupils. As this is current research, I point out that the LAMap has to be seen as the first step in our analysis of how teachers organize a target song and guide the pupils to achieve the goal. As subsequent methodological steps, we use the LAMap to contextualize the teacher’s statements made in the interview while watching the lesson. Thus, we combine the graphic overview of the event with the issues that arise in the interview (Savona, 2021). In this way, we account for a teacher’s activities while teaching and their various statements, which naturally include self-evaluations, judgments, and visions of future professional development. By using the LAMap methodology to transcribe lessons in the longitudinal study on the professional development of pre-service generalist teachers, we obtained a comprehensive overview of the course of each individual case.

So far, we have discovered a tendency in the pre-service teachers to shift attention from instructional issues to more musical ones with respect to not only their own performances and song transformations but also the quality of children’s productions. We have also found changes in their verbal reflections: for instance, an increase and differentiation of the vocabulary to describe and evaluate song-related activities.

## Conclusion

In the present article, I have endeavored to conceptualize song transmission as a cultural practice, its informal and formal versions, and emphasized normativity as an inherent property that has far-reaching consequences for evaluation, judgment, and justification. Whereas song transmission as a practice may function well without any investment of extra effort in its analysis, a scientific perspective aims to render explicit the norms and rules that guide it. I have tried to demonstrate the need for this endeavor by reporting applications of unspoken and open-ended criteria to evaluate and judge generalist teachers’ work. With no existing consensus regarding some of the rules and norms applicable to characterizing formal song transmission and its efficiency, I have argued that this cultural practice remains unsatisfactory from the point of view of achieving convincing educational and aesthetic judgments and justifications. Therefore, I have proposed applying a structural and descriptive approach and a didactic perspective to study this process and underlying regularities systematically. Didactics, as an emerging scientific field, provides certain concepts that help in structuring the phenomena at stake; in the present case, these are teaching a song to pupils and achieving a structural similarity in subsequent performances. Teacher, pupils, knowledge, and their interrelations are the main focus of didactic research. These components, I claim, are also valid units to employ in studying formal song transmission.

As a secondary emphasis, I have made the case for making explicit the norms and rules that govern formal song transmission. To this end, I have proposed a Children’s Song Grammar that allows the song to be conceptualized as a regularly structured entirety or unit. The grammar permits explicit statements and criteria regarding the well-formedness of a vocal utterance on the one hand, and of song models as abstract ideas on the other. Hence, the outlined version of this grammar represents an attempt to systematize and render explicit some of the rules inherent in song and its transmission. As its normative nature is neither verifiable nor falsifiable, its validity must be negotiated with regard to, for instance, culturally specific traditions, changeable conventions, and ways of formal representation.

Finally, as proof of principle, I have described methodological and empirical research on formal song transmission, first by focusing on a child constructing her vocal expression adaptively toward a given song model. This case illustrated her strategies, one consisting in recalling her idea of a familiar song, which she expresses; however, it seemed to be a spontaneous tonal invention. Second, drawing on current research on generalist teachers’ song leading, I presented our methodology for transcribing the recurrent and constitutive activities that take place during a lesson at school. The aim is to gain a comprehensive view of teachers’ activities, such as monitoring, as well as self-evaluations and self-judgments, in order to reconstruct their professional development as song leaders in class.

Overall, I have attempted to support the argument that research on formal song transmission, a professional capacity of generalist teachers, ought to aim to make explicit the inherent normativity of this practice by applying a structure-genetic and didactic view of the phenomena. Although this statement is itself normative, I am convinced that the scientific strategy I advocate here promises to increase the systematicity of research in this field (Hoyningen-Huene, 2013).

## Data Availability Statement

The raw data used for this article are video recordings. They are not available in order to protect the anonymity of the participants. Questions regarding the datasets should be directed to Stefanie Stadler Elmer, stefanie.stadlerelmer@uzh.ch. Figures 4A–F are created at the basis of acoustic data from videos using two software programs, Pitch Analyzer and Notation Viewer. These programs were developed by the theoretical physicist Franz-Josef Elmer. They are freely available at: http://mmatools.sourceforge.net/ (Accessed October 30, 2021).

## Ethics Statement

Ethical review and approval were not required for the study on human participants in accordance with the local legislation and institutional requirements. Written informed consent to participate in this study was provided by the participant pre-service teachers and teachers, and by the children’s parents.

## Author Contributions

SSE conceptualized the studies, collected all data, produced Figures 1–4, organized the finances for the projects, and wrote the article. Figures 5 and 6 were produced together with Annamaria Savona, PhD student, supervised by the author.

## Funding

This research was supported by the Swiss National Science Foundation; project “The Song Leading Capacity – Developing Professionalism in Teacher Education (So-Lead, Nr. 100019_179182)” and the Schwyz University of Teacher Education.

## Conflict of Interest

The author declares that the research was conducted in the absence of any commercial or financial relationships that could be construed as a potential conflict of interest.

## Publisher’s Note

All claims expressed in this article are solely those of the authors and do not necessarily represent those of their affiliated organizations, or those of the publisher, the editors and the reviewers. Any product that may be evaluated in this article, or claim that may be made by its manufacturer, is not guaranteed or endorsed by the publisher.

## Footnotes

^1^François Joliat, Annamaria Savona, Gabriella Cavasino, Anna Hürlimann, Armin Wyrsch.

^2^I use the term *synphonize* according to the Greek meaning to *sound together*. The term *synchronous* refers to simultaneity, and the term *polyphonic* to several voices singing together.

^3^The present version is a slight revision of the original one published in German language (Stadler Elmer, 2015). It is also partly published in English in Stadler Elmer (2020).

^4^In this context, I avoid the term “rhythm” because “there are so many meanings for this term that it would be useless to try to enumerate them all.” Arom (1991, p. 186). They have no common denominator

^5^“Chain songs” (Kettenlieder) are songs with minor changes and infinite verses that allow enumeration of things such as activities, colors, or names.

^6^Annamaria Savona, Gabriella Cavasino, Olivia Fündeling, Ana María Camiña Montero, and Anna Hürlimann.

## References

[ref1] Abler W. L. (1989). On the particulate principle of self-diversifying systems. J. Soc. Biol. Struct. 12, 1–13.

[ref2] Arom S. (1991). African Polyphony and Polyrhythm: Musical Structure and Methodology. Cambridge: Cambridge University Press.

[ref3] Brandom R. (1994). Making It Explicit: Reasoning, Representing, and Discursive Commitment. Cambridge: Harvard University Press.

[ref4] Broome J. (2018). Reason Fundamentalism and What Is Wrong With It. *Vol*. 1. ed. Star D. (Oxford, UK: Oxford University Press).

[ref5] Chevallard Y. Bosch M. (2020). “Anthropological theory of the didactic (ATD),” in Encyclopedia of Mathematics Education. ed. Lerman S. (Berlin/Heidelberg: Springer), 53–61.

[ref6] de Lacy P. V. (2006). Markedness: Reduction and Preservation in Phonology. Cambridge Studies in Linguistics 112. Cambridge, New York: Cambridge University Press.

[ref7] Deutsch D. Henthorn T. Lapidis R. (2011). Illusory transformation from speech to song. J. Acoust. Soc. Am. 129, 2245–2252. doi: 10.1121/1.3562174, PMID: 21476679

[ref8] Dowling W. J. (1984). “Development of musical schemata in children’s spontaneous singing,” in Cognitive Processes in the Perception of Art. eds. Crozier W. R. Chapman A. J. (Amsterdam: Elsevier Science Publishers, North-Holland), 145–162.

[ref9] Dowling P. (2020). “Recontextualization in Mathematics Education,” in Encyclopedia of Mathematics Education. ed. Lerman S. (Berlin/Heidelberg: Springer), 717–721.

[ref10] Ewen C. J. van der Hulst H. (2001). The Phonological Structure of Words: An Introduction. Cambridge, MA: Cambridge University Press.

[ref11] Feyerabend P. K. (1975). Against Method: Outline of an Anarchistic Theory of Knowledge. London: New Left Books.

[ref12] Goldsmith J. A. (1990). Autosegmental and Metrical Phonology. Oxford, UK: B. Blackwell.

[ref13] Hall T. A. (2000). Phonologie: Eine Einführung. Berlin: de Gruyter.

[ref14] Hennessy S. (2017). Approaches to increasing the competence and confidence of student teachers to teach music in primary schools. Education 45, 1–12. doi: 10.1080/03004279.2017.1347130

[ref15] Hoyningen-Huene P. (2013). Systematicity: The Nature of Science. Oxford, UK: Oxford University Press.

[ref16] Jordania J. (2011). Why Do People Sing? Music in Human Evolution. Tbilisi: LOGOS.

[ref17] Kager R. (2007). “Feet and metrical stress,” in The Cambridge Handbook of Phonology. ed. de Lacy P. (Cambridge, MA: Cambridge University Press), 195–227.

[ref18] Kuhn T. S. (1962). The Structure of Scientific Revolutions. Chicago: University of Chicago Press.

[ref19] Langer S. K. (1948). Philosophy in a New Key. A Study in the Symbolism of Reason, Rite, and Art. New York: The New American Library.

[ref20] Langer S. (1953). Feeling and Form: A Theory of Art Developed from Philosophy in a New Key. London: Routledge & Kegan Paul.

[ref21] Lerdahl F. Jackendoff R. (1983). A Generative Theory of Tonal Music. Cambridge, MA: MIT Press.

[ref22] Liao M.-Y. Campbell P. S. (2014). An analysis of song-leading by kindergarten teachers in Taiwan and the USA. Music. Educ. Res. 16, 144–161. doi: 10.1080/14613808.2013.851661

[ref23] Liao M.-Y. Campbell P. S. (2016). Teaching children’s songs: a Taiwan–US comparison of approaches by kindergarten teachers. Music. Educ. Res. 18, 20–38. doi: 10.1080/14613808.2015.1049256

[ref24] Lindblom B. Sundberg J. (2007). “The human voice in speech and singing,” in Springer Handbook of Acoustics. ed. Rossing T. D. (Berlin/Heidelberg: Springer), 669–717.

[ref25] Merker B. (2002). Music: The missing Humboldt system. Music. Sci. 6, 3–21. doi: 10.1177/102986490200600101

[ref27] Merker B. (2005). The conformal motive in birdsong, music, and language: An introduction. Ann. N. Y. Acad. Sci. 1060, 17–28. doi: 10.1196/annals.1360.003, PMID: 16597746

[ref28] Merker B. (2006). The uneven interface between culture and biology in human music [commentary]. Music. Percept. 24, 95–98. doi: 10.1525/mp.2006.24.1.95

[ref29] Merker B. (2009). “Ritual foundations of human uniqueness,” in Communicative Musicality. eds. Malloch S. Trevarthen C. (Oxford, UK: Oxford University Press), 45–59.

[ref30] Merker B. (2012). “The vocal learning constellation: Imitation, ritual culture, encephalization,” in Music, Language and Human Evolution. ed. Bannan N. (Oxford, UK: Oxford University Press), 215–260.

[ref32] Merker B. (2015). Seven theses on the biology of music and language. Signata 6, 195–213. doi: 10.4000/signata.1081

[ref67] Merker B. (2018). “When extravagance impresses: recasting esthetics in evolutionary terms,” in The Oxford Handbook of Music and the Brain. eds. Thaut M. H. Hodges D. A. (Oxford, UK: Oxford University Press).

[ref33] Merker B. Morley I. Zuidema W. (2015). Five fundamental constraints on theories of the origins of music. Philos. Trans. R. Soc. B 370:20140095. doi: 10.1098/rstb.2014.0095, PMID: 25646518 PMC4321136

[ref34] Merker B. Morley I. Zuidema W. (2018). “Five fundamental constraints on theories of the origins of music,” in The Origins of Musicality. ed. Honing H. (Cambridge, MA: MIT Press), 70–104. 10.1098/rstb.2014.0095 PMC4321136 25646518

[ref26] Meyer M. (2009). Sprachliche Wohlgeformtheit. Eine kritische Bestandsaufnahme. [Linguistic well-formdness. A critical discourse]. Zeitschrift für Sprachwissenschaft 28, 14–150.

[ref35] Morley I. (2013). The Prehistory of Music: Human Evolution, Archaeology, and the Origins of Musicality. Oxford, UK: Oxford University Press.

[ref36] Nettl B. (2000). “An Ethnomusicologist contemplates universals in musical sound and musical culture,” in The Origins of Music. eds. Wallin N. L. Merker B. Brown S. (Cambridge, MA: The MIT Press), 463–472.

[ref37] Papoušek M. Papoušek H. (1981). Musical elements in the infant’s vocalization: Their significance for communication, cognition, and creativity. Adv. Infancy Res. 1, 163–224.

[ref38] Papoušek H. Papoušek M. (1987). “Intuitive parenting: A dialectic counterpart to the infant’s integrative competence,” in Handbook of Infant Development. ed. Osofsky J. D. (Hoboken, NJ: Wiley), 669–720.

[ref39] Patel A. D. (2008). Music, Language, and the Brain. Oxford, UK: Oxford University Press.

[ref40] Plümacher M. (1999). “Wohlgeformtheitsbedingungen für Bilder,” in Bildgrammatik. Interdisziplinäre Forschungen zur Syntax bildlicher Darstellungsformen [Grammar of pictures. Interdisciplinary research on the syntax of representations]. eds. Sachs-Hombach K. Rehkämper K. (Schiedam, Netherlands: Scriptum), 47–56.

[ref41] Povel D.-J. (2010). Melody generator: a device for algorithmic music construction. J. Softw. Eng. Appl. 3, 683–695. doi: 10.4236/jsea.2010.37078

[ref42] Rebuschat P. Rohrmeier M. Hawkins J. A. Cross I. (2012). Language and Music as Cognitive Systems. Oxford, UK: Oxford University Press.

[ref43] Roth (2016). “What would it be to be a norm?” in Normativity and Naturalism in the Philosophy of Social Sciences. ed. Risjord M. (Oxfordshire, UK: Routledge, Taylor & Francis Group), 43–59.

[ref44] Rouse J. (2007). Social Practices and Normativity. Philos. Soc. Sci. 37, 46–56. doi: 10.1177/0048393106296542

[ref45] Rouse J. (2015). Articulating the World: Conceptual Understanding and the Scientific Image. Chicago: University of Chicago Press.

[ref46] Santa M. (2019). Hearing Rhythm and Meter: Analyzing Metrical Consonance and Dissonance in Common-Practice Period Music. Oxfordshire, UK: Routledge.

[ref47] Savona A. (2021). “Analysing lesson-based interviews using the Lesson Activities Map (LAMap) as a visual tool.” in *Education and New Developments. World Institute for Advanced Research and Science (WIARS)*. ed. Carmo M. (Portugal). 472–476. Available at: http://end-educationconference.org/proceedings/ (Accessed October 30, 2021).

[ref48] Savona A. Stadler Elmer S. Hürlimann A. E. Joliat F. Cavasino G. (2021). The lesson activities map: a domain-specific lesson transcription methodology. Eurasian J. Educ. Res. 10, 705–717. doi: 10.12973/eu-jer.10.2.705

[ref49] Schatzki T. (1996). Social Practices: A Wittgensteinian Approach to Human Activity and the Social. Cambridge, UK: Cambridge University Press.

[ref50] Schneuwly B. (2020). « Didactique »? Didactique 1, 40–60. doi: 10.37571/2020.0103

[ref51] Schneuwly B. (2021). “‘Didactiques’ is not (entirely) ‘Didaktik’. The origin and atmosphere of a recent academic field,” in Didaktik and Curriculum in Ongoing Dialogue. eds. Krogh E. Qvortrup A. Graf S. T. (Oxfordshire, UK: Routledge), 164–184.

[ref52] Stadler Elmer S. (2002). Kinder singen Lieder: Über den Prozess der Kultivierung des vokalen Ausdrucks. [Children‘s Singing – On the Process of Cultivating Vocalization] Waxmann. Available at: https://www.zora.uzh.ch/id/eprint/94783/1/02_Stadler_Kinder_singen_Lieder.pdf (Accessed October 30, 2021).

[ref53] Stadler Elmer S. (2015). Kind und Musik. [Child and Music]. Berlin/Heidelberg: Springer.

[ref54] Stadler Elmer S. (2020). “From canonical babbling to early song singing and its relations to speech,” in The Routledge Companion to Interdisciplinary Studies in Singing. *Vol*. 1. eds. Russo F. Ilari B. Cohen A. J. (Oxfordshire, UK: Routledge), 25–38.

[ref55] Stadler Elmer S. Elmer F. J. (2000). A new method for analyzing and representing singing. Psychol. Music 28, 23–42. doi: 10.1177/0305735600281003

[ref56] Stadler Elmer S. Weniger L. (2019). Gestalt und Wohlgeformtheit: Kinder zeichnen Raumkörper. AER 17 e-journal der swissarteducation.ch. Available at: https://sfkp.ch/artikel/raumlich-zeichnen (Accessed November 28, 2019).

[ref57] Stumpf C. (1911). Die Anfänge der Musik [The origins of music]. Barth. Nachdruck (1979). Hildesheim, Germany: Georg Olms Verlag.

[ref58] Sundberg J. Lindblom B. (1976). Generative theories in language and music descriptions. Cognition 4, 99–122. doi: 10.1016/0010-0277(76)90011-1

[ref59] Swain N. Bodkin-Allen S. (2017). Developing singing confidence in early childhood teachers using acceptance and commitment therapy and group singing: a randomized trial. Res. Stud. Music Educ. 39, 109–120. doi: 10.1177/1321103X17700141

[ref60] Tierney A. Patel A. D. Breen M. (2018). Acoustic foundations of the speech-to-song illusion. J. Exp. Psychol. Gen. 147, 888–904. doi: 10.1037/xge0000455, PMID: 29888940

[ref61] Turner S. P. (1994). The Social Theory of Practices: Tradition, Tacit Knowledge and Presuppositions. Cambridge, MA: Polity Press.

[ref68] Valsiner J. (2020). Sensuality in Human Living: The Cultural Psychology of Affect. Cham: Springer.

[ref62] van der Hulst H. (eds.) (2014). “The study of word accent and stress: past, present, and future,” in Word Stress – Theoretical and Typological Issues. Cambridge, MA: Cambridge University Press, 3–55.

[ref63] von Ehrenfels C. (1890). Über Gestaltqualitäten. [On Gestalt qualities] Vierteljahrsschrift für wissenschaftliche Philosophie, Jg. 13, 249–292. Available at: https://phaenomenologica.de/?p=139 (Accessed October 30, 2021).

[ref64] von Humboldt W. (1836). *Über die Verschiedenheit des menschlichen Sprachbaus und ihren Einfluss auf die geistige Entwicklung des Menschengeschlechts*. Berlin: Druckerei der Königlichen Akademie der Wissenschaften.

[ref65] von Wright G. H. (1963). Norm and Action. A Logical Inquiry. Oxfordshire, UK: Routledge and Kagan Paul.

[ref66] Wertheimer M. (1923). Untersuchungen zur Lehre von der Gestalt. Psychol. Forsch. 4, 301–350. doi: 10.1007/BF00410640

